# Comprehensive Coagulation Management in Cardiac Surgery: Anticoagulants, Heparin Resistance, Monitoring, and Bleeding Risks: Coagulation Management in Cardiac Surgery

**DOI:** 10.31661/gmj.v14i.3981

**Published:** 2025-11-05

**Authors:** Sohrab Negargar, Elnaz Javanshir

**Affiliations:** ^1^ Cardiovascular Research Center of Tabriz University of Medical Sciences, Tabriz, Iran

**Keywords:** Cardiopulmonary Bypass, Blood Coagulation, Anticoagulants, Heparin, Viscoelastic Testing

## Abstract

Effective coagulation management is pivotal to optimizing outcomes in cardiac surgery, influencing bleeding risk, transfusion requirements, and overall perioperative safety. This comprehensive review examines current strategies, limitations, and emerging innovations across anticoagulant use, coagulation monitoring, and bleeding management. Unfractionated heparin remains the standard for cardiopulmonary bypass (CPB) anticoagulation, owing to its rapid reversibility, though challenges such as heparin resistance persist. Alternatives including low molecular weight heparins, direct thrombin inhibitors, and novel oral anticoagulants are reserved for select indications and carry specific limitations. Perioperative coagulation monitoring is essential; tools such as activated clotting time (ACT), anti-factor Xa assays, and viscoelastic tests (e.g., thromboelastography [TEG] and rotational thromboelastometry [ROTEM]) guide targeted therapy. Despite these advances, bleeding remains common due to factors including preoperative antithrombotic therapy, CPB-induced coagulopathy, and postoperative hemostatic deficits. Management strategies center on prophylactic antifibrinolytics, individualized transfusion protocols, and the judicious use of reversal agents. Emerging frontiers including machine learning–enhanced viscoelastic algorithms, targeted antithrombotics (e.g., factor XI inhibitors), AI-based bleeding prediction, and gene therapy for inherited coagulopathies promise to personalize and improve care. Continued research is warranted to validate novel therapies and refine evidence-based protocols for coagulation management in cardiac surgery.

## Introduction

Cardiac surgery often involves major bleeding and profound hemostatic perturbations, so that meticulous coagulation management is critical to patient outcomes [1]. Indeed, cardiovascular operations frequently require perioperative transfusion (on the order of 43-54% of isolated CABG and 54-67% of valve cases) [2][3], and any such transfusion is associated with substantially higher mortality (e.g. a 6.9-fold increase in in-hospital death) [1]. Cardiopulmonary bypass (CPB) itself provokes systemic inflammation and contact activation, triggering a consumptive coagulopathy; if anticoagulation is inadequate, this can cause life-threatening clotting, while excessive anticoagulation or dysregulated fibrinolysis leads to serious bleeding and thromboembolism [4]. Complicating matters, many cardiac surgical patients are already on antithrombotic therapy (e.g. aspirin, P2Y12 inhibitors or warfarin) before surgery [5]. More recently, the rising use of direct oral anticoagulants (DOACs) in patients with atrial fibrillation and venous thromboembolism has added complexity, since DOACs lack simple point-of-care tests and until recently had no readily available antidotes [6]. These factors together make the management of anticoagulants in cardiac surgery inherently complex [5].

Intraoperatively, unfractionated heparin (UFH) remains the standard anticoagulant for CPB, typically given as a 300-400 IU/kg bolus to achieve an ACT of roughly 400-480 seconds [7]. However, response to heparin varies greatly between patients. An estimated 4-26% of adults exhibit heparin resistance (HR) defined as failure to reach target ACT despite high heparin dosing [8]. HR is often due to low antithrombin levels or other factors that blunt heparin’s effect. When HR occurs, management becomes more difficult: clinicians may give extra heparin, antithrombin concentrates, or switch to alternative anticoagulants, but these measures themselves can increase bleeding risk if not carefully monitored [4][8]. Thus, identifying HR in advance (for example via heparin dose-response assays or antithrombin activity tests) is important to tailor the anticoagulation strategy and minimize complications [8]. In parallel, careful coagulation monitoring is essential throughout surgery. In addition to routine ACT measurements and standard coagulation panels, many centers now use point-of-care viscoelastic testing (e.g. TEG/ROTEM) and platelet-function assays to assess global hemostasis during and after CPB [5]. Such algorithmic, viscoelastic-guided management has been shown to significantly reduce blood product transfusion and blood loss in major surgery [9]. Achieving a careful balance between bleeding and clotting risks remains the ultimate goal in cardiac surgery. Excessive perioperative bleeding is strongly associated with increased complications, morbidity, and mortality [10]. Conversely, inadequate anticoagulation or inappropriate heparin reversal can lead to catastrophic thrombotic events, including circuit clotting, myocardial infarction, and stroke [6][10]. Therefore, a comprehensive coagulation strategy incorporating personalized anticoagulant management, vigilant monitoring, and timely hemostatic interventions is crucial to optimize patient outcomes in cardiac surgery [5][10].

This review article aimed to provide a comprehensive overview of current strategies, clinical challenges, and recent advances in coagulation management during cardiac surgery. Specifically, it addresses the roles and limitations of various anticoagulants, approaches to coagulation monitoring, identification and management of bleeding risks, and emerging therapeutic modalities.

## Physiology of Coagulation in Cardiac Surgery

Hemostasis is classically described by the intrinsic (contact) and extrinsic (tissue factor) coagulation pathways, which converge on a final common pathway leading to thrombin generation and fibrin clot formation [11]. Tissue injury exposes tissue factor (TF) to plasma, triggering the extrinsic pathway as TF binds factor VIIa to activate factor X and trace amounts of thrombin [12]. Simultaneously, damage to endothelium and contact with subendothelial collagen can initiate the intrinsic pathway via factor XII activation (contact activation), although factor XII is not essential for normal hemostasis in vivo [13]. Thrombin is a key mediator of coagulation, enhancing clot formation even at low levels by activating factors V, VIII, and XI, and by potently stimulating platelets through protease-activated receptors [14]. This propagation phase occurs on the surface of activated platelets, accelerating the assembly of coagulation factor complexes and precipitating a burst of thrombin sufficient to convert fibrinogen to fibrin and stabilize the clot [12]. Regulatory mechanisms are concurrently engaged to localize clotting; for example, excess thrombin feeds back to activate the protein C pathway, which inactivates factors Va and VIIIa to attenuate further thrombin generation. The end result is a tightly controlled fibrin clot that seals injured vessels while limiting thrombosis in the surrounding circulation [13][14].

## Impact of Cardiopulmonary Bypass (CPB) on Coagulation Pathways

CPB imposes multiple derangements on coagulation physiology, often resulting in a diffuse coagulopathy at the end of cardiac surgery [5][12]. Blood contact with the non-endothelial surfaces of the CPB circuit activates the contact system, triggering factor XII (Hageman factor) and the intrinsic coagulation pathway [15]. Factor XIIa generated by this contact not only initiates the clotting cascade but also produces bradykinin and activates complement, contributing to systemic vasodilation and inflammation [16]. Surgical tissue trauma and inflammatory mediators can concurrently activate the extrinsic (tissue factor) pathway, so that ultimately both pathways converge to upregulate the common pathway and thrombin generation during CPB[15]. Notably, substantial thrombin and factor Xa generation can occur despite full heparinization, as evidenced by detectable thrombin activity during bypass [17]. At the same time, CPB causes hemodilution and consumptive losses of coagulation factors (including prothrombin and fibrinogen) and of platelets, which significantly impairs the blood’s thrombin-generating capacity [12]. The large heparin doses used for CPB add a pharmacologic anticoagulation effect and also raise levels of tissue factor pathway inhibitor (TFPI), a potent inhibitor of the initiation phase of coagulation that remains elevated even after protamine reversal [18]. Fibrinolytic pathways are likewise stimulated by CPB; endothelial release of tissue plasminogen activator (tPA) and other factors can cause excessive fibrinolysis, manifesting as high D-dimer levels and contributing to bleeding risk [5]. Indeed, prophylactic antifibrinolytic therapy (e.g. tranexamic acid) is now standard in cardiac surgery to counteract CPB-induced hyperfibrinolysis [5]. In aggregate, the coagulopathy of CPB is multifactorial and involves a combination of coagulation factor dilution/consumption, thrombin inhibition, and accelerated fibrin clot breakdown. These disruptions help explain why patients emerging from CPB are at high risk of diffuse oozing and bleeding complications if not properly managed [5][12].

## Inflammatory Response and Platelet Dysfunction

CPB induces a systemic inflammatory response that is tightly coupled with coagulation dysfunction. Blood exposure to the non-endothelial surfaces of the CPB circuit activates the complement cascade and releases cytokines and kinins, triggering leukocyte activation and endothelial injury [5][15]. Pro-inflammatory mediators, including tumor necrosis factor-α and interleukin-1, rise significantly during CPB, promoting capillary leak, tissue edema, and organ dysfunction [19]. In parallel, thrombin generation exacerbates inflammation by upregulating endothelial adhesion molecules (e.g., P-selectin, E-selectin), fostering neutrophil adhesion and perpetuating what has been termed "thromboinflammation" [15][17].

This inflammatory state is a key contributor to the platelet dysfunction observed during and after CPB. Platelets are activated and degranulated upon exposure to the bypass circuit, leading to receptor shedding, aggregation, and eventual clearance [5]. As a result, postoperative thrombocytopenia is common, with platelet counts often falling by 40-60% due to hemodilution, sequestration, and consumption [20]. Moreover, even retained platelets exhibit impaired aggregation due to shear stress and inflammatory inhibition [12].

While some functional recovery occurs postoperatively as inflammatory mediators subside, platelet dysfunction persists for several hours after protamine administration [21]. In prolonged or complex surgeries, this dysfunction is more profound, with platelets often coated with fibrin or trapped in microthrombi, rendering them hemostatically ineffective [12][22].

## Anticoagulants in Cardiac Surgery

Effective anticoagulation is fundamental in cardiac surgery, particularly during CPB, to prevent catastrophic thrombosis in the extracorporeal circuit. For over half a century, unfractionated heparin (UFH) has remained the standard systemic anticoagulant for CPB in both adult and pediatric patients [23]. In recent years, however, alternative anticoagulant classes and new oral anticoagulants have assumed important roles in perioperative management [24]. Table-1 compared the key features of anticoagulants.

## Unfractionated Heparin (UFH) – Standard of Care

**Figure-1:**
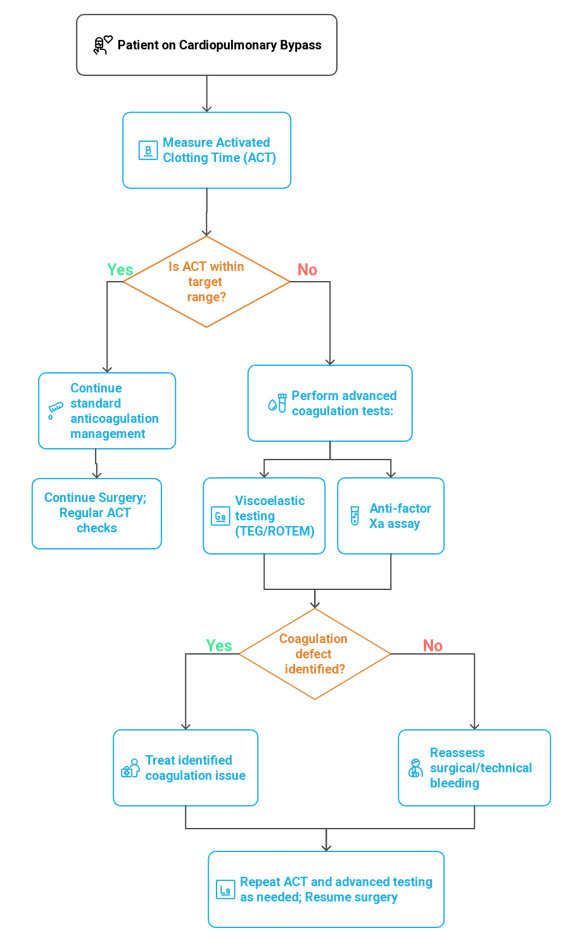
Figure 1. Flowchart of Coagulation Monitoring in Cardiac Surgery

**Figure-2:**
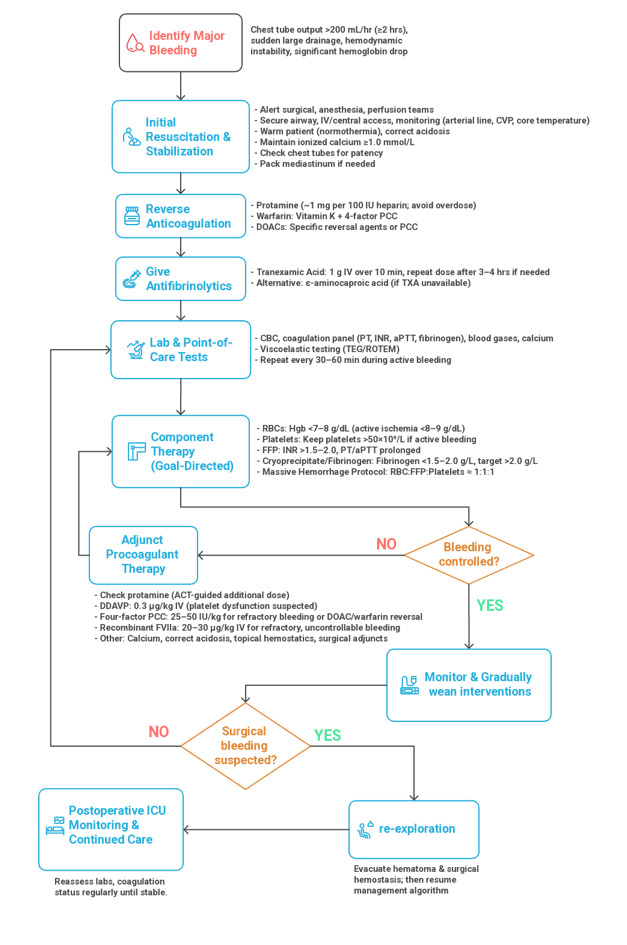
Figure 2. Algorithm for Management of Major Bleeding in Cardiac Surgery

**Table:** Table1. Anticoagulant Comparison in Cardiac Surgery

Feature	UFH ** (Unfractionated Heparin) **	LMWH ** (e.g., Enoxaparin) **	Bivalirudin ** (Direct Thrombin Inhibitor) **	DOACs/NOACs ** (e.g., Apixaban, Rivaroxaban) **
Mechanism of Action	Enhances AT activity to inhibit factor Xa and IIa	Preferential inhibition of factor Xa via AT	Direct reversible thrombin (IIa) inhibitor	Direct inhibition of Xa (or IIa for dabigatran)
CPB Compatibility	Yes- gold standard for CPB	Not suitable during CPB	Used in CPB when heparin is contraindicated	Not suitable for CPB (limited use)
Monitoring	ACT, anti-Xa levels	Anti-Xa	ACT, possibly aPTT; specialized assays (ECT, DTI assay)	PT/INR, anti-Xa (limited POC applicability)
Reversibility	Fully reversible with protamine	Partially reversible with protamine	No specific reversal (short half-life)	Dabigatran: idarucizumab; Xa inhibitors: andexanet
Half-life	~1-2 hrs (IV)	~4-6 hrs (SC)	~25 min (normothermia); longer in hypothermia	~12-17 hrs (varies by agent and renal function)
Use in HIT	Contraindicated	Cross-reactive	Preferred alternative	Does not cause HIT
Dosing Route	IV (bolus and infusion)	SC	IV infusion (weight-based)	Oral
Dosing in renal impairment	independent of renal function	Need dose adjustment and avoided if CrCl <30 mL/min	Only bivalirudin dosing must be adjusted	Need dose adjustment and Dabigatran is contraindicated in CrCl <30 mL/min
Onset of Action	Immediate (IV)	~2-4 hours	Immediate	1-4 hours after ingestion
Advantages	Inexpensive, fast onset, fully reversible, well-established for CPB	Convenient outpatient use, less monitoring	Works independently of AT, no HIT, predictable effect	Fixed dosing, no routine monitoring, good long-term safety
Limitations	Variable response (requires AT), risk of HIT, rebound effect	Cannot be used in CPB, partially reversible, accumulates in renal impairment	No reversal agent, clot risk in stagnant flow, costly	Not used intraoperatively, reversal agents limited, bleeding risk if not stopped early

Clinical Use: UFH is the mainstay anticoagulant for patients undergoing cardiac surgery with CPB [26]. In adult cardiac surgery, a weight-based bolus of UFH (typically ~300-400 U/kg) is administered before cannulation, and adequate anticoagulation is confirmed by an activated clotting time (ACT)>480 seconds prior to initiating bypass [23]. Additional heparin doses are given as needed to maintain the ACT in the target range during surgery [23]. Pediatric cardiac surgery also relies on UFH for CPB, but children (especially infants) often exhibit unique challenges - extreme hemodilution on bypass and developmentally low antithrombin levels can blunt heparin’s effect [27]. Consequently, children may require higher weight-indexed heparin dosing and antithrombin supplementation to reach target ACT. Despite these differences, UFH with ACT monitoring remains the gold standard anticoagulation technique for both adult and pediatric CPB [23][27].

Advantages: The enduring dominance of UFH is due to several key advantages. UFH has a rapid onset and short half-life, allowing fine control of anticoagulation during surgery [28]. It is highly effective at inhibiting thrombin and factor Xa when bound to antithrombin, and decades of experience have made its behavior well-characterized in the surgical setting. UFH is also inexpensive and widely available [4][8]. Crucially, it has a specific antidote: protamine sulfate rapidly and (in the case of UFH) completely neutralizes heparin’s anticoagulant effect [4]. This reversibility permits safe termination of CPB and surgical hemostasis once the procedure is finished. Additionally, UFH’s efficacy can be monitored in real time by point-of-care tests (ACT), which is essential for managing anticoagulation during complex procedures [23]. These qualities - effective anticoagulation with quick on/off and a reliable reversal agent - make UFH uniquely well-suited as the default anticoagulant in cardiac surgery [28].

Limitations: UFH is not without drawbacks. Its pharmacokinetic and pharmacodynamic profile is variable; patients can exhibit heparin resistance, defined as an unexpectedly low ACT response to a standard dose. A recent analysis found heparin resistance in roughly 17-20% of adult cardiac surgeries (often defined as failure to reach ACT ≥480 s after 500 U/kg of UFH) [23][29]. This variability is often due to acquired antithrombin deficiency, elevated heparin-binding proteins, or high plasma volume states [23]. Management strategies include additional heparin dosing, administering antithrombin concentrate or fresh frozen plasma, or (if necessary) switching to an alternative anticoagulant [8][23]. Another significant limitation is the risk of heparin-induced thrombocytopenia (HIT), an immune-mediated prothrombotic reaction. HIT complicates approximately 1-2% of cardiac surgery patients exposed to UFH [30]. An additional concern is that protamine, while an effective reversal agent, can itself cause adverse reactions severe hypotension, bradycardia, and even anaphylactoid reactions or pulmonary hypertension in susceptible individuals [31]. Recent guidelines continue to endorse individualized UFH dosing and monitoring (e.g., heparin concentration assays or ACT-based protocols) as essential for safe CPB [32].

Reversal: The reversal of UFH is achieved with protamine sulfate, which is routinely administered at the end of CPB to restore normal coagulation. Protamine (a positively charged polypeptide) binds the anionic heparin, forming a stable inactive complex. Typically, full heparin neutralization is obtained within minutes of protamine administration [4].

In modern practice, protamine is dosed based on the amount of heparin given (often ~1-1.3 mg protamine per 100 U heparin, adjusted to ACT or heparin concentration if using a titration assay) [4]. If residual anticoagulation is suspected (continued bleeding with prolonged ACT), additional protamine can be given, though excess protamine itself can paradoxically impair coagulation. No other anticoagulant discussed has a reversal agent as universally used and as immediately effective as protamine is for heparin [4][8].

## Low Molecular Weight Heparin (LMWH)

Clinical Use: Low molecular weight heparins (e.g., enoxaparin, dalteparin) are fragments of heparin with more selective anti-factor Xa activity [33]. LMWH is not routinely used as the primary anticoagulant during CPB because its longer half-life and partial irreversibility make it less controllable in the intraoperative setting. UFH is preferred for on-pump surgery. However, LMWH has important roles in the perioperative period of cardiac surgery [26].

LMWH is commonly employed for bridging and thromboprophylaxis. For example, a patient on chronic warfarin (for a mechanical valve or atrial fibrillation) may be transitioned to LMWH before surgery when warfarin is held - particularly if they are high thromboembolic risk [34]. Guidelines support bridging with UFH or LMWH in high-risk patients (e.g., mechanical valve in mitral position or recent stroke) when interrupting warfarin for surgery [34]. Postoperatively, LMWH is frequently used for prophylaxis against venous thromboembolism after cardiac surgery (started 24-48 hours after surgery, if bleeding is controlled) [35].

Advantages: Compared to UFH, LMWH has a more predictable dose-response and a longer plasma half-life, permitting once or twice daily dosing without continuous infusion [4]. It does not require routine laboratory monitoring in most patients, which is convenient for outpatient prophylaxis or bridging[36].

Another advantage is the significantly lower incidence of HIT with LMWH. HIT can still occur, but is far less common on the order of <1% in surgical patients (e.g., ~0.2-0.5% incidence, versus a few percent with UFH) [37]. From a practical standpoint, subcutaneous administration is a double-edged sword: it spares the patient continuous IV access, which is particularly advantageous in outpatient settings or in children once central lines are removed [36].

Limitations: LMWH, while convenient for outpatient use, presents significant limitations in the intraoperative setting. Its prolonged half-life and incomplete reversibility by protamine pose challenges in managing acute surgical bleeding [38]. Real-time monitoring is impractical, as LMWH is not detectable by ACT and anti-Xa assays are not point-of-care [26]. Additionally, renal clearance necessitates caution in patients with kidney dysfunction, and subcutaneous administration can be painful and logistically difficult in pediatric populations. Infants may require higher weight-based dosing, complicating standard protocols [39][40]. LMWH is also contraindicated in patients with HIT due to potential cross-reactivity. These factors restrict its use mainly to pre- or postoperative periods rather than during surgery [41].

Reversal: As noted, protamine sulfate can be used to partially reverse LMWH. Standard practice is to give protamine if a patient on therapeutic LMWH has an unexpected need for surgery or if there is bleeding, dosing 1 mg protamine per 100 anti-Xa units of LMWH [42][43].This will typically neutralize the majority of LMWH’s antifactor IIa activity and some portion of anti-Xa activity, but an anti-Xa effect will persist. Repeat protamine doses can further reduce anti-Xa activity, but complete reversal is not achievable with protamine alone [42].

## Direct Thrombin Inhibitors (e.g., Bivalirudin)

Clinical Use: Direct thrombin inhibitors (DTIs) bind and inhibit thrombin without needing antithrombin as a cofactor. In cardiac surgery, the most prominent DTI is bivalirudin, a short-acting thrombin inhibitor [44]. These agents are primarily used only when heparin is contraindicated, such as in patients with a history of HIT or heparin allergy [45]. Bivalirudin has been successfully employed as the anticoagulant for CPB in patients with acute HIT, allowing life-saving cardiac operations to proceed without heparin [44]. Because bivalirudin is not cleared by the liver or kidneys to a significant, it remains active in the circuit until metabolized or diluted/removed [44][45].

Advantages: Bivalirudin, a direct thrombin inhibitor, is a valuable alternative when heparin is contraindicated, such as in HIT [44]. It acts independently of antithrombin, inhibits both free and clot-bound thrombin, and does not trigger HIT[23]. With a short half-life (~25 min) and no need for reversal, its effect tapers rapidly after infusion stops facilitating postoperative hemostasis [45]. Unlike heparin, it causes less platelet activation, making it potentially gentler during CPB. Clinical data, including pediatric cases, suggest comparable safety and efficacy to heparin, even in complex surgeries like heart transplants and VAD placement [44][45].

Limitations: Despite their utility in heparin contraindication, DTIs like bivalirudin have major limitations. Lack of a reversal agent, risk of thrombosis during blood stasis, and complex intraoperative management limit their use [44]. Monitoring is less precise than with UFH-ACT can be unreliable at high concentrations, and specific assays are not readily available [46][47]. Cost is significantly higher than UFH, and metabolism slows during hypothermia or low cardiac output, prolonging effect [34]. Experience with DTIs in CPB is limited, especially in pediatrics, and guidelines recommend them only when heparin is absolutely contraindicated [47].

Reversal: There is no specific antidote for bivalirudin or other direct thrombin inhibitors. Management relies on their short half-life and supportive measures [45].

After stopping infusion, bivalirudin is cleared by enzymatic breakdown and dilution rewarming and good perfusion accelerate this. In urgent cases, hemofiltration or dialysis may help, though not consistently. Supportive strategies include antifibrinolytics, blood products, and, in emergencies, PCCs or recombinant factor VIIa (off-label) [48]. Experimental agents like ciraparantag and andexanet alfa are under investigation but not approved. The lack of reliable reversal underscores the need for meticulous planning when using DTIs in cardiac surgery [49].

## New Oral Anticoagulants (NOACs)

Clinical Use: Direct oral anticoagulants (NOACs/DOACs), including factor Xa inhibitors (rivaroxaban, apixaban, edoxaban) and dabigatran (a thrombin inhibitor), are now common in patients presenting for cardiac surgery [50]. Although not used during CPB, their perioperative management is critical [6]. For elective surgery, NOACs are usually held 2-5 days pre-op, depending on renal function and bleeding risk. Bridging is rarely needed [51].

Residual NOACs can interfere with ACT monitoring, risking under-heparinization during CPB. Special caution is needed if NOAC clearance is incomplete, with clotting assays or thromboelastography aiding assessment [52].

Importantly, NOACs are contraindicated in mechanical heart valves due to increased thrombotic risk. Warfarin remains standard in this group [53].

Advantages: NOACs offer multiple advantages over warfarin that are relevant to surgical planning. Their predictable pharmacokinetics allow fixed dosing without routine monitoring, simplifying management [54]. They have a quick onset (1-4 hours) and short half-lives (~12-17 hours), enabling shorter preoperative interruption without bridging in most cases [51].

Compared to warfarin, NOACs are associated with a lower risk of intracranial bleeding and fewer drug or dietary interactions [55]. This improves safety, particularly if residual anticoagulant is present. They also enhance compliance, especially in younger patients, and can often be resumed 1-3 days post-op without bridging [56].

Finally, NOACs do not trigger heparin-induced thrombocytopenia (HIT), making intraoperative use of UFH safe even after prior NOAC therapy [54].

Limitations: Despite their advantages, NOACs present several perioperative challenges. Unlike warfarin, they lack routine monitoring tools—standard labs (PT, aPTT) may appear normal despite active anticoagulation, and specific assays (anti-Xa, ecarin) are not widely available or rapid. This complicates urgent surgery when NOAC levels are uncertain. Also, reversal options are limited [56].Moreover, Renal impairment prolongs drug clearance, increasing bleeding risk [57]. Cost and lack of use in mechanical valves remain barriers [53].

Reversal Strategies: To briefly recap the specific reversal strategies available for NOACs (since this is critical knowledge for perioperative management):

• Idarucizumab rapidly reverses dabigatran [58].

• Andexanet alfa reverses factor Xa inhibitors but is costly and not always available [56].

• 4F-PCC is often used off-label when specific antidotes are unavailable [57].

## Heparin Resistance in Cardiac Surgery

### Definition and Clinical Relevance

Heparin resistance (HR) is the failure to achieve adequate anticoagulation—typically an ACT>480 seconds despite standard dosing of unfractionated heparin (UFH)[32]. In cardiac surgery, this poses serious risks including circuit thrombosis and bleeding due to excessive dosing. HR affects 4-26% of adults, with much higher rates in pediatrics, especially neonates, due to low antithrombin (AT) levels [4][8].

### Mechanisms and Risk Factors

The primary cause of HR is AT deficiency, whether congenital or more commonly acquired (e.g. from prior heparin use, sepsis, liver dysfunction) [4]. Heparin’s efficacy depends on AT; low AT activity (<70-80%) reduces its anticoagulant effect [59]. Additional contributors include heparin-binding proteins, elevated fibrinogen/factor VIII, low albumin, and inflammatory states such as infective endocarditis [60]. Certain medications, like andexanet alfa, can also provoke HR by disrupting the heparin-AT interaction [61].

### Diagnosis

HR should be suspected if ACT remains subtherapeutic after appropriate heparin dosing. Confirmation involves:

• Anti-Xa levels (therapeutic heparin concentration despite low ACT) [8],

• AT activity assays (typically <70% in HR) [62],

• Heparin dose-response tests [29], and

• TEG/ROTEM (heparinase comparison) [4].

### Management

Initial steps include re-dosing heparin. However, when AT deficiency is confirmed or suspected, AT concentrate is the treatment of choice, offering rapid correction without volume overload [8]. If unavailable, FFP provides AT replacement but in larger volumes [62]. If ACT remains subtherapeutic despite these measures, bivalirudin may be used as an alternative anticoagulant [23]. It provides AT-independent anticoagulation and is effective for CPB, though lacks a reversal agent. Argatroban is a secondary option, reserved for rare cases when both UFH and bivalirudin are contraindicated [4][23]. HR demands a proactive, multidisciplinary approach, especially in high-risk groups. Early recognition, appropriate testing, and targeted treatment primarily AT supplementation are key to maintaining safe anticoagulation and avoiding surgical complications [8].

## Monitoring Coagulation in Cardiac Surgery

Effective coagulation monitoring is essential in cardiac surgery to balance anticoagulation during CPB and manage bleeding post-bypass. A multimodal approach including ACT, anti-factor Xa assays, viscoelastic testing (TEG/ROTEM), and point-of-care (POC) devices offers both safety and specificity across diverse patient populations [26][27]. Figure-1 illustrated the flowchart of coagulation monitoring.

### Activated Clotting Time (ACT)

ACT is the standard intraoperative test for monitoring UFH during CPB. It provides rapid, bedside feedback and is widely used in both adults and pediatrics. A target ACT>480 seconds is typically maintained [63]. However, ACT is affected by hypothermia, hemodilution, and antithrombin levels, limiting its reliability in certain patients, especially neonates. In such cases, heparin concentration monitoring or antithrombin supplementation may be necessary [27].

### Anti-factor Xa Assays

Anti-Xa testing is the gold standard for heparin quantification, reflecting actual drug levels. It is primarily used when ACT is unreliable, such as in heparin resistance or complex pediatric cases [63]. Despite its accuracy, routine intraoperative use is limited by turnaround time and laboratory dependency. Emerging POC systems may enhance its intraoperative feasibility [8].

### TEG and ROTEM (Viscoelastic Testing)

Viscoelastic Testing offer real-time, whole-blood assessment of clot dynamics. These tools identify the mechanism of bleeding, whether platelet dysfunction, fibrinogen deficiency, or hyperfibrinolysis, and guide targeted transfusion [64]. Meta-analyses confirm reduced bleeding and transfusion with viscoelastic-guided algorithms, and STS guidelines endorse their use (Class I recommendation) [51].

### Point-of-care Coagulation Tools

Modern POC devices allow bedside assessment of PT/INR, aPTT, fibrinogen, and platelet function in minutes [65]. These tools complement ACT and TEG/ROTEM, accelerate clinical decision-making, and reduce blood loss [66]. Limitations include cost, calibration requirements, and inter-device variability [63].

## Bleeding Risks in Cardiac Surgery

**Table:** Table2. Risk Factors for Major Bleeding After Cardiac Surgery

**Preoperative Factors **	**Intraoperative Factors **	**Postoperative Factors **
Recent use of antiplatelet or anticoagulants agents (e.g. clopidogrel ≤5 days pre-op)	Prolonged CPB time	High chest tube output (>200 mL/hr in adults; >5-10 mL/kg/hr in children)
Anemia (Hgb <12 g/dL)	Hypothermia during CPB (e.g. <28°C)	Coagulopathy (e.g. low fibrinogen, platelet dysfunction)
Thrombocytopenia or known platelet dysfunction	Heparin-protamine imbalance	• Residual anticoagulant effect (e.g. delayed DOAC clearance)
Liver dysfunction or inherited coagulopathy	Dilutional coagulopathy from excessive crystalloid	Heparin rebound after protamine
Chronic kidney disease	CPB-induced fibrinolysis	• Post-CPB fibrinolysis (e.g. hyperfibrinolysis not treated with antifibrinolytics)
Reoperation or prior sternotomy	Surgical complexity (e.g. reoperation, multiple valve procedures, aortic surgery)	• Inadequate correction of coagulopathy (e.g. missed hypofibrinogenemia or low platelet count)
Pediatric considerations (immature coagulation system in neonates and infants)	Poor intraoperative hemostasis (e.g. diffuse microvascular bleeding at closure)	• Delayed reintervention for surgical bleeding

Bleeding remains a major perioperative challenge in cardiac surgery, impacting both adult and pediatric populations. It is associated with increased morbidity, transfusion requirements, and mortality [10]. Risk factors span the preoperative, intraoperative, and postoperative periods and require coordinated, evidence-based management strategies [64]. Table-2 summarized the risk factors for major bleeding in cardiac surgery.

### Preoperative Risk Factors

• Antithrombotic Medications: Recent use of P2Y12 inhibitors or DOACs increases bleeding risk. Guideline-directed discontinuation and, if needed, reversal (e.g., idarucizumab, andexanet) are critical for urgent cases [25].

• Anemia and Coagulopathy: Preoperative anemia is common and increases transfusion risk. Treating iron deficiency and optimizing hemoglobin reduces bleeding and improves outcomes. Coagulopathies (e.g., liver disease, uremia) also heighten risk [25].

• Renal Dysfunction and Redo Surgery: CKD impairs platelet function and prolongs drug clearance. Reoperations increase bleeding due to adhesions and surgical complexity [67].

• Pediatrics: Neonates have immature coagulation systems and low blood volume, leading to high transfusion needs. Preoperative optimization (e.g., correcting anemia, vitamin K status) is essential [64].

### Intraoperative Risks

• CPB-Induced Coagulopathy: Hemodilution, platelet activation, and factor consumption during CPB reduce clotting capacity. Fibrinogen, platelet count, and function fall significantly[10]. Risk is amplified in infants due to high circuit-to-blood-volume ratios [5].

• Hypothermia and Surgical Complexity: Hypothermia impairs coagulation; longer, complex procedures increase CPB time and blood loss [68]. Strategies include minimizing bypass duration and using antifibrinolytics (e.g., tranexamic acid) [69].

• Heparin-Protamine Imbalance: Inadequate reversal causes bleeding; excess protamine also impairs coagulation. Titrated dosing and ACT monitoring ensure proper balance [32].

• Platelet Dysfunction: CPB impairs platelet adhesion and aggregation. Platelet transfusion is often required, especially in neonates. TEG/ROTEM help guide transfusion decisions [5].

### Postoperative Complications

• Chest Tube Output: Excessive or rising chest tube drainage may signal surgical bleeding or coagulopathy. Thresholds vary (e.g., >200 mL/hr in adults, >5 mL/kg/hr in children) and should prompt timely intervention [70].

• Transfusion Thresholds: Restrictive transfusion practices (e.g., Hb <7-8 g/dL) reduce complications. Pediatric thresholds vary by physiology (e.g., Hb ≥9 g/dL in single-ventricle palliation) [71].

• Thrombo-Hemorrhagic Balance: Balancing bleeding and thrombosis is crucial, especially in valve and shunt-dependent patients. Post-op anticoagulation timing is individualized based on bleeding stability [32].

### Outcomes and Quality Improvement

Bleeding and transfusion are tracked quality metrics linked to worse outcomes. Protocol-driven blood management, adherence to STS/SCA and EACTS/EACTA guidelines, and the use of POC monitoring, antifibrinolytics, and multidisciplinary protocols reduce transfusions and improve safety [25][32].

## Management of Bleeding in Cardiac Surgery

Effective bleeding control in cardiac surgery relies on a combination of pharmacologic agents, targeted transfusion strategies, anticoagulant reversal, and, when necessary, surgical re-exploration. Management should be goal-directed, based on clinical assessment and coagulation testing (e.g. TEG/ROTEM) [25]. Figure-2 demonstrated an algorithm for management of major bleeding in cardiac surgery.

### Pro-hemostatic Pharmacologic Agents

• Antifibrinolytics: Tranexamic acid (TXA) is standard in both adult and pediatric protocols. It significantly reduces bleeding and transfusion needs, though high doses may increase seizure risk [32].

• Desmopressin (DDAVP): Enhances platelet function via vWF release. Reserved for uremia or CPB-induced platelet dysfunction [25].

• Recombinant Factor VIIa: A last-resort therapy for refractory bleeding. Effective in select cases, but carries thrombotic risk and is not routinely recommended [25].

### Blood Component Therapy

• RBCs: Restrictive transfusion thresholds (Hgb ≥7-8 g/dL) are recommended. Pediatric targets may vary, especially in neonates or cyanotic lesions [71].

• Platelets: Indicated if platelet count <50×109/L or if function is impaired. Pediatric cases often require transfusion despite normal counts due to qualitative dysfunction [72].

• FFP: Used for coagulopathy with INR >1.5-2.0 or guided by POC coagulation tests [72].

• Cryoprecipitate: Given for fibrinogen <1.5-2.0 g/L or low clot strength on TEG/ROTEM. Fibrinogen concentrate is an alternative with similar efficacy [73].

### Reversal Agents

• Protamine: Standard reversal for UFH (1 mg per 100 U heparin). Must be titrated to avoid over- or under-correction [74].

• Vitamin K: Used with PCC/FFP to reverse warfarin. IV administration offers delayed but sustained effect [75].

• PCCs: Rapidly correct warfarin-induced coagulopathy and may be used for refractory bleeding. Dosing is weight-based (25-50 IU/kg) [72].

• DOAC Reversal: Idarucizumab (dabigatran) and andexanet alfa (Xa inhibitors) are used emergently; PCCs are second-line if specific agents are unavailable [58].

### Surgical Re-exploration

• Indicated when chest tube output exceeds thresholds (e.g., >200 mL/hr in adults, >10 mL/kg/hr in children) or hemodynamic instability suggests surgical bleeding [76].

• Early re-exploration (<6 hours post-op) is preferred to reduce complications (e.g., tamponade, coagulopathy) [77].

• Surgical goals include control of bleeding sites, clot evacuation, and adjuncts like fibrin sealants. Medical hemostatic therapy continues as needed post-repair [78]. Bleeding management in cardiac surgery demands an integrated, multidisciplinary approach combining preventive strategies, real-time monitoring, and evidence-based interventions. Optimizing coagulation while minimizing transfusion and avoiding delays in surgical correction is essential to improving outcomes [6].

## Future Directions and Emerging Therapies

Advances in personalized coagulation monitoring and targeted therapies are transforming bleeding management in cardiac surgery [79]. One of the most promising developments is the integration of viscoelastic testing with machine learning (ML) [80]. TEG and ROTEM are already well-established tools for assessing dynamic clot function, but next-generation models aim to embed these assays within real-time decision-support systems [81]. Recent studies have demonstrated that machine learning can codify expert ROTEM interpretations, enabling automated, data-driven transfusion algorithms [82]. For instance, ensemble ML models trained on preoperative and intraoperative variables have been shown to accurately predict post-CPB fibrinogen and prothrombin levels [83]. These tools, often described as "super-learner" systems, may soon be integrated into anesthesia workstations or perfusion consoles, offering clinicians real-time coagulation forecasts and transfusion guidance [84].

Gene therapy represents a paradigm shift in the surgical management of inherited bleeding disorders [85]. The novel gene therapies including valoctocogene roxaparvovec (Roctavian) and etranacogene dezaparvovec (Hemgenix) significantly decrease bleeding events and prophylactic factor infusions in hemophilia A and B [86]. Patients receiving these therapies maintain stable factor levels for years, reducing or eliminating the need for perioperative factor replacement [87]. As a result, many gene-treated hemophilia patients can undergo cardiac surgery using standard anticoagulation protocols, without the intensive hemostatic support previously required [88]. These therapies have not only normalized bleeding risk in a subset of patients with previously severe coagulopathy but also represent a model for future genetic treatments in rare bleeding disorders [89].

Together, these innovations are reshaping the approach to bleeding and coagulation in cardiac surgery. From predictive analytics and AI-guided transfusion to targeted and gene-based therapies, the future of coagulation management is increasingly personalized, data-driven, and safer for even the most complex surgical patients [81].

## Conclusion

Comprehensive coagulation management in cardiac surgery involves an intricate balance of anticoagulation, bleeding control, and meticulous hemostasis monitoring. Advances in anticoagulant strategies, targeted hemostatic therapies, and point-of-care coagulation assessments have significantly improved perioperative outcomes, reducing bleeding complications and transfusion requirements. Key takeaways from this review emphasize that effective management of coagulation requires precise patient-specific strategies, such as individualized anticoagulant dosing, personalized viscoelastic monitoring, and tailored use of pro-hemostatic agents based on validated thresholds and algorithms.

These approaches are now becoming standardized through clinical guidelines and enhanced by technological advancements, including artificial intelligence-driven predictive models and novel targeted antithrombotic therapies.

Equally crucial is the recognition that optimal coagulation management in cardiac surgery relies on robust multidisciplinary collaboration among cardiac surgeons, anesthesiologists, perfusionists, intensivists, and hematologists. Such integrated teamwork ensures early recognition and prompt management of bleeding risks, improves surgical and pharmacological decision-making, and enhances patient safety and outcomes.

Despite considerable progress, gaps in knowledge remain, particularly concerning individualized anticoagulation protocols, pediatric-specific guidelines, and emerging gene therapies relevant to patients with hereditary coagulopathies undergoing cardiac surgery. Future research should prioritize randomized controlled trials evaluating the clinical impact and cost-effectiveness of personalized coagulation monitoring systems, the role of novel targeted anticoagulants, and AI-based predictive analytics. Addressing these knowledge gaps through rigorous scientific inquiry will continue to improve coagulation management strategies, ultimately translating into better clinical outcomes and resource utilization in cardiac surgery.

## Conflict of Interest

None.
